# Posterior Reversible Encephalopathy Syndrome in Peritoneal Dialysis Patients: A Four-Case Series

**DOI:** 10.3390/jcm15052003

**Published:** 2026-03-05

**Authors:** Nanda Shajan, Gabrielė Mikšytė, Diana Sukackienė, Giedrė Žulpaitė, Raminta Lukšaitė-Lukštė, Laurynas Rimševičius, Marius Miglinas

**Affiliations:** 1 Faculty of Medicine, Vilnius University, M. K. Ciurlionio 21, 03101 Vilnius, Lithuania; 2 Clinic of Gastroenterology, Nephrourology and Surgery, Institute of Clinical Medicine, Faculty of Medicine, Vilnius University, M. K. Ciurlionio 21, 03101 Vilnius, Lithuania; 3 Department of Radiology, Nuclear Medicine and Medical Physics, Institute of Biomedical Sciences, Faculty of Medicine, Vilnius University, 03101 Vilnius, Lithuania

**Keywords:** end-stage renal disease, peritoneal dialysis, posterior reversible encephalopathy syndrome

## Abstract

**Background/Objectives:** Posterior reversible encephalopathy syndrome (PRES) is a neurological condition characterized by acute neurological symptoms and vasogenic edema, usually affecting the posterior circulation. It is described in end-stage renal disease (ESRD), but its presentation in peritoneal dialysis (PD) is not well defined. We aimed to describe the clinical, radiological, and dialysis-related features of PRES in PD patients and highlight factors relevant for diagnosis and management. **Materials and Methods:** We conducted a retrospective descriptive case series of four ESRD patients on PD or recently transitioned from PD to hemodialysis (HD) who developed PRES at a single center. Clinical data, laboratory results, dialysis characteristics, and neuroimaging findings were obtained from medical records. PRES was diagnosed based on acute neurological symptoms in the setting of severe hypertension and uremia, with CT and/or MRI findings supportive of PRES when present and exclusion of alternative diagnoses. **Results:** All patients presented with acute neurological manifestations, including headache, visual disturbances, seizures, and/or altered consciousness, in the context of marked hypertension and uremia. Neuroimaging findings ranged from normal CT/MRI to subtle bilateral occipital hypodensities and, in one case, extensive supra- and infratentorial vasogenic edema with internal hydrocephalus and subependymal edema. In three patients, inadequate volume or solute control on PD prompted temporary or permanent transition to HD to improve blood pressure and fluid management. With antihypertensive therapy, seizure control when required, correction of metabolic disturbances, and optimization of dialysis, all patients recovered clinically, with time to PRES resolution ranging from 7 to 43 days. **Conclusions:** PRES should be considered in PD patients with new-onset seizures, visual symptoms, or unexplained changes in mental status, particularly during hypertensive crises and uremia. Early CT/MRI, prompt blood pressure control, and careful adjustment of dialysis modality appear important for achieving favorable neurological outcomes.

## 1. Introduction

Posterior reversible encephalopathy syndrome (PRES) is a neurological disorder characterized by acute onset of headache, seizures, altered mental status, nausea, and visual disturbances, typically accompanied by bilateral vasogenic edema predominantly affecting the parieto-occipital regions [1,2]. PRES is associated with various clinical and iatrogenic triggers. Although its pathophysiology is not fully understood, it most often occurs with acute elevations in blood pressure and is linked to chronic hypertension, renal failure, eclampsia, transplantation with immunosuppression, sepsis, autoimmune disease, and cytotoxic drugs [2,3,4]. The syndrome was first described in 1996 as reversible posterior leukoencephalopathy syndrome (RPLS) by Granata et al., based on observations in 15 patients with characteristic clinical and radiological features [5]. PRES affects a wide age range, from children to older adults, and appears to be more prevalent in females [3,5]. However, the prevalence of PRES in dialysis populations remains ambiguous due to a lack of systematic studies. Available reports suggest that PRES is rare in patients with ESRD, with an estimated prevalence of approximately 0.8%, although it is likely under-recognized [6].

Patients undergoing peritoneal dialysis (PD) often exhibit chronic volume overload, hypertension, and metabolic disturbances [7,8]. Persistent uremia may further contribute to endothelial dysfunction [9]. Because the rapidity of blood pressure elevation, rather than the absolute value alone, appears to be critical for PRES development [3,5], this combination of volume overload, poorly controlled hypertension, and uremia may increase cerebral vulnerability in PD patients and predispose them to PRES. However, current evidence on PRES in dialysis patients is limited to small case-based reports or series, and the interaction between PD-specific factors or chronic kidney disease itself and classical PRES triggers remains poorly characterized [10,11].

When promptly recognized and managed, the clinical manifestations of PRES usually resolve within about one week, and magnetic resonance imaging (MRI) abnormalities improve over the following days to weeks [12]. Given the increasing awareness of PRES, documenting individual cases is essential for improving our understanding of its risk factors, clinical presentation, and diagnostic challenges. By presenting these cases of PRES in patients undergoing peritoneal dialysis, we aim to highlight the complexity of PRES diagnosis in this population and emphasize the importance of early intervention in optimizing patient outcomes. Therefore, the objective of this study was to describe in detail the clinical, radiological, and dialysis-related features of PRES in PD patients and to explore practical factors that may assist clinicians in recognizing, diagnosing, and managing this condition.

## 2. Materials and Methods

### 2.1. Literature Search

A focused literature search was performed in PubMed (MEDLINE) and Google Scholar using the keywords “posterior reversible encephalopathy syndrome”, “PRES”, “dialysis”, “peritoneal dialysis”, and “peritoneal dialysis complications”. Only English-language case reports, case series, and review articles published up to September 2025 were included to contextualize the findings of this case series.

### 2.2. Study Design and Case Identification

This retrospective descriptive case series includes four patients with ESRD who developed PRES while receiving PD or hemodialysis (HD) at Vilnius University Hospital Santaros Clinics (Vilnius, Lithuania) between February 2022 and February 2025.

Cases were identified retrospectively from the electronic medical records of the nephrology department, dialysis unit, and intensive care unit by screening for PD patients (or those recently switched from PD to HD) who experienced acute neurological symptoms compatible with PRES in the setting of ESRD, with supportive neuroimaging findings when available.

We included adult patients (≥18 years) with ESRD who were on PD and who developed an acute neurological syndrome compatible with PRES, for whom sufficient clinical and neuroimaging data were available. Patients were excluded if the acute neurological event was primarily attributed to another diagnosis (such as ischemic or hemorrhagic stroke, central nervous system infection, or primary central nervous system vasculitis) or if clinical documentation and imaging were incomplete.

### 2.3. Data Collection

Clinical data were obtained through direct patient interviews and review of available medical records. Extracted variables included demographic characteristics, underlying renal disease, comorbidities, dialysis modality and duration, dialysis-related complications, presenting neurological symptoms, blood pressure at admission, laboratory findings, neuroimaging results, treatments, and short-term outcomes. The study was conducted in accordance with the principles of the Declaration of Helsinki. According to the institutional policy of Vilnius University Hospital Santaros Clinics, retrospective case series based on anonymized clinical data do not require formal approval from the regional ethics committee. Written informed consent for publication was obtained from all patients.

### 2.4. Definition of PRES

Episodes were classified as PRES when all of the following criteria were fulfilled: (i) acute onset of at least one major neurological symptom (seizure, visual disturbance, severe headache with encephalopathy, or altered mental status); (ii) presence of severe hypertension and/or uremia in the setting of ESRD on dialysis; (iii) brain CT and/or MRI without an alternative structural explanation for the acute event (such as large-vessel ischemic stroke, intracranial hemorrhage, central nervous system infection, or space-occupying lesion); and (iv) complete or near-complete clinical recovery after blood pressure optimization and correction of metabolic disturbances. When CT or MRI showed findings compatible with vasogenic edema, the episode was considered “definite PRES”; when imaging was normal or nonspecific, but all other criteria were met, the diagnosis was considered PRES. All cases were reviewed by the treating ICU doctors in collaboration with nephrologists, radiologists, and, when involved, the consulting neurologist.

## 3. Case Presentations

### 3.1. Case 1

A 28-year-old woman with ESRD secondary to IgA nephropathy, managed with automated peritoneal dialysis (APD) for the previous 15 months, presented to the emergency department with altered consciousness and disorientation. Her past medical history was notable for secondary arterial hypertension (Grade II) and hypertensive cardiopathy. On admission, her blood pressure was 150/90 mmHg (Table 1).

Shortly after admission, the patient experienced a generalized tonic–clonic seizure, with no prior history of epilepsy, and was promptly transferred to the intensive care unit (ICU).

Brain MRI was performed because PRES was suspected based on the clinical presentation; however, no radiological abnormalities were detected. Despite the absence of MRI changes, the overall clinical picture in the setting of ESRD on APD was consistent with a diagnosis of PRES.

The patient was treated with intravenous antihypertensive agents, anticonvulsant therapy, and supportive measures. Due to progressive worsening of uremia and inadequate clearance with peritoneal dialysis, temporary hemodialysis was initiated. She received 11 hemodialysis sessions, which resulted in progressive stabilization of her neurological and metabolic status.

After 28 days of intensive care and multidisciplinary management, the patient showed significant clinical improvement, with normalization of laboratory parameters. In accordance with her lifestyle preferences and long-term treatment goals, a plan was made to transition back to automated peritoneal dialysis.

### 3.2. Case 2

A 20-year-old female with ESRD secondary to X-linked Alport syndrome had been managed with APD for 11 months. She presented to the emergency department with acute-onset bilateral blurred vision, severe headache, nausea, and vomiting. Her past medical history was notable for primary arterial hypertension.

On admission, her blood pressure was markedly elevated at 200/120 mmHg (Table 1), and an acute cerebrovascular event was suspected. Non-contrast head CT with CT angiography (CTA) showed no infarction, perfusion defect, or intracranial hemorrhage, but revealed subtle bilateral low-attenuation changes in the occipital subcortical white matter, raising the possibility of PRES; no cervical or intracranial arterial occlusion or significant stenosis was seen. She was transferred to the ICU for further management.

Twelve hours later, brain MRI showed no visible pathological changes; compared with the initial CT, this was interpreted as a favorable evolution of the previously noted edema. In the context of her characteristic clinical presentation and risk profile, PRES was diagnosed in the setting of ESRD on APD.

Antihypertensive therapy was initiated to achieve gradual blood pressure control. Given inadequate solute clearance and volume control, temporary hemodialysis was performed. Once her metabolic parameters stabilized, peritoneal dialysis was resumed. Over the course of seven days, the patient experienced complete resolution of her visual disturbances and headaches, with full recovery of neurological function. Peritoneal dialysis was subsequently continued without complications.

### 3.3. Case 3

A 32-year-old man with a history of ESRD secondary to juvenile nephronophthisis had been receiving dialysis since 2004. He underwent kidney transplantation in 2006. In 2021, he experienced kidney transplant rejection, after which PD was re-initiated because of a progressive decline in graft function.

The patient presented to the emergency department with severe weakness, headache, diffuse body pain, and acute-onset delirium. On admission, his blood pressure was critically elevated at 210/116 mmHg (Table 1). Given the clinical suspicion of hypertensive encephalopathy, he was promptly transferred to the ICU, where deep sedation was required for neurological stabilization.

On admission, PRES was clinically suspected, and a non-contrast head CT with CT angiography was performed. No acute focal lesions, hemorrhage, or perfusion defects were detected (ASPECTS score 10), and no thrombi were seen in the cervical or intracranial arteries. Five days later, repeat head CT again showed no acute density changes, hemorrhage, or evidence of intracranial arterial or venous sinus occlusion, but revealed left maxillary sinusitis and bilateral otomastoiditis. MRI could not be performed because the examination requires prolonged time in the scanner and the patient was too hemodynamically unstable due to septic shock from pneumonia.

Management included aggressive antihypertensive therapy, which was initially resistant to treatment, anticonvulsant therapy, supportive care, and continued dialysis. Over the following weeks, gradual clinical improvement was observed. After 43 days of multidisciplinary care, the patient’s neurological status stabilized, blood pressure was controlled, and his renal replacement therapy regimen was optimized.

### 3.4. Case 4

A 41-year-old male with ESRD secondary to primary focal segmental glomerulosclerosis (FSGS) had been managed with APD for one month. He presented to the emergency department with a severe occipital headache, dizziness, and resistant hypertension. During hospitalization, due to persistent uncontrolled blood pressure, he developed a generalized seizure accompanied by blurred vision. His past medical history was significant for primary arterial hypertension.

**Figure 1:**
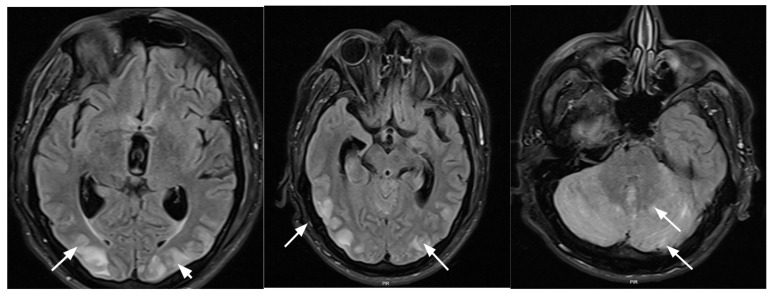
Brain MRT T2 FLAIR axial plane. Hyperintense subcortical zones are seen in the posterior part supra- and infratentorially (arrows).

On admission, his blood pressure was critically elevated at 187/114 mmHg (Table 1). CT and MRI of the brain demonstrated both supratentorial and infratentorial changes characteristic of PRES, as well as signs of internal hydrocephalus and subependymal edema (Figure 1).

The patient was transferred to the ICU for close monitoring and management. Antihypertensive therapy and symptomatic treatment were initiated. Given inadequate volume control and persistent hypertension, renal replacement therapy was transitioned from peritoneal dialysis to hemodialysis.

Over 22 days, the patient’s neurological condition stabilized, and his PRES-related visual disturbances resolved completely. Blood pressure remained well controlled on maintenance hemodialysis, and no further seizures were observed.

Table 1 describes the clinical profiles of the four patients, highlighting their demographics, dialysis adequacy, and medication needs. Young to middle-aged adults (aged 20–41) exhibit severely elevated uremic markers, with creatinine levels reaching up to 1497 µmol/L. Suboptimal uremia control is seen despite the use of APD. The standard clinical targets for PD adequacy are a minimum weekly Kt/V of 1.7 and a weekly creatinine clearance of at least 45 L per 1.73 m^2^. It was not reached in Case 1, and the Case 4 patient was hypervolemic. Also, a significant finding is the usage of polypharmacy for blood pressure management, particularly in Case 3, requiring nine different antihypertensives. Overall, the data shows high cardiovascular and metabolic burden.

Table 2 details the clinical cases where chronic kidney disease and arterial hypertension triggered PRES. At the onset of the syndrome, the patients exhibited severe neurological symptoms like seizures, disorientation, and visual disturbances, coinciding with high blood pressure peaks up to 210/116 mmHg. While Case 4 showed extensive vasogenic edema on MRI, other cases had no visible lesions, highlighting that PRES can be a clinical diagnosis. Recovery was marked by the stabilization of blood pressure, though the time to resolution varied significantly from 7 to 43 days. This data emphasizes the critical role of blood pressure management in resolving neurological complications in kidney patients.

## 4. Discussion

In this single-center retrospective case series, we describe four patients with ESRD on PD who developed PRES. All patients presented with acute neurological symptoms—headache, visual disturbances, seizures, or altered mental status—in the context of severe hypertension, uremia, and, in some cases, infection or inadequate volume control. These cases illustrate how classical PRES mechanisms, namely, hypertensive autoregulatory failure and endothelial dysfunction, interact with dialysis-specific factors such as chronic volume overload, metabolic derangements, and dialysis adequacy in PD patients.

Previous reports have described only isolated cases of PRES in adults and children receiving PD, suggesting that this presentation is rare but likely under-recognized in the ESRD population [11,13]. Common themes across these reports include the rapid onset of neurological symptoms and the high correlation with hypertensive crises. Differences lie in the atypical localizations (e.g., frontal lobe or brainstem) seen in pediatric cases [14] and sometimes specific dialysis modality, but this is disputable. In a series of three patients described by Oliveira, individuals were young, had uncontrolled hypertension and most presented shortly after PD-induction [15]. Some other analyzed reports demonstrate that PRES in the PD population is frequently associated with specific metabolic and hemodynamic disruptions, such as severe volume overload [16], acute hypertension [17], and even rare complications like pleuroperitoneal communication [18], which is specific to PD itself. Findings from different cases highlight those systemic triggers—including sepsis-induced endothelial dysfunction [19] or drug-induced differentiation syndrome [20]—can exacerbate the breakdown of the blood–brain barrier in uremic patients.

According to a review by Moreiras-Plaza, who analyzed 15 previously published case reports, PD can act as an indirect cause due to fluid overload and uncontrolled arterial hypertension. He suggests that PD might even act as a protective factor due to gradual fluid removal compared to HD [10]. Our series builds upon the existing literature by providing a comparative analysis of four PD-associated PRES episodes in adult patients with distinct underlying renal diseases, comorbidities, and clinical courses.

### 4.1. Pathophysiology

PRES is generally thought to result from a failure of cerebral autoregulation leading to vasogenic edema. Two main mechanisms are discussed: hypertensive autoregulatory failure and endothelial dysfunction [5].

Cerebrovascular autoregulation normally maintains relatively stable cerebral blood flow despite changes in systemic blood pressure [4]. According to the hyperperfusion theory, a rapid and severe rise in blood pressure can overwhelm these mechanisms, causing cerebral hyperperfusion, breakdown of the blood–brain barrier (BBB), and extravasation of plasma and proteins, which in turn leads to vasogenic edema. The posterior brain regions are particularly vulnerable because of relatively sparse sympathetic innervation, which helps explain the typical parieto-occipital predominance on imaging. In some cases, extreme hypertension may also trigger vasospasm and local ischemia, further damaging the BBB [1,3]. In our series, all four patients presented with marked hypertension (150/90–210/116 mmHg). Although Case 1 had comparatively lower values, they were clearly above her usual range in the context of chronic ESRD, where autoregulatory reserve is already limited. Cases 2, 3, and 4 had severe hypertensive crises, consistent with the hyperperfusion model. These observations align with reports indicating that both absolute blood pressure levels and the rate of elevation are key triggers for PRES in patients with kidney disease [1,3,21,22].

Hypertension is common and often poorly controlled in ESRD. In PD, slower ultrafiltration rates and more liberal fluid intake can lead to persistent or subclinical hypervolemia [21]. In this setting, even modest or rapid rises in blood pressure may exceed the patient’s reduced autoregulatory capacity, predisposing to PRES. In our cohort, three patients (Cases 1, 2, and 4) ultimately required transition from PD to HD during the acute episode, suggesting that PD was, at least transiently, insufficient to control volume status and blood pressure.

The second major mechanism proposes that PRES is driven by endothelial dysfunction caused by circulating endogenous or exogenous toxins. This model helps explain why up to 30% of patients with PRES do not exhibit the severe hypertension typical of the hyperperfusion theory [4,5]. In patients with PD, chronic uremia, recurrent infections, sustained volume overload, and prolonged exposure to bioincompatible dialysate may cause persistent low-grade inflammation with increased expression of pro-inflammatory and pro-fibrotic mediators (e.g., VEGF, TGF-β, and AGEs/RAGE), leading to systemic endothelial dysfunction [23,24]. In our series, Cases 1, 2, and 4 had marked uremia and electrolyte disturbances, while Case 3 developed bacterial pneumonia and septic shock. These heterogeneous triggers all converge on endothelial injury and BBB disruption, supporting the coexistence and interaction of hypertensive and endothelial mechanisms in PD-related PRES.

In summary, hypertensive autoregulatory failure and endothelial dysfunction represent complementary pathways in PRES pathogenesis, and in PD patients they are likely to coexist and mutually reinforce each other.

### 4.2. Clinical Imaging

In acute neurological presentations, CT is usually the first imaging modality. Although less sensitive than MRI for detecting PRES, it can show hypoattenuation in affected white matter and is essential for excluding intracranial hemorrhage or large territorial infarction. When present, CT findings in PRES typically reflect bihemispheric vasogenic edema with a predominance in the parieto-occipital regions [4].

MRI is the gold standard for diagnosing PRES because of its superior sensitivity and anatomical resolution [25]. The typical pattern consists of symmetric, reversible T2-weighted and FLAIR hyperintensities corresponding to subcortical vasogenic edema, most commonly in the parieto-occipital lobes, and MRI without contrast usually allows differentiation from alternative diagnoses such as posterior circulation infarction or hypoxic–ischemic injury [4,5,25].

The imaging findings in our patients ranged from normal studies to extensive PRES-typical changes. In Case 1, brain MRI performed soon after symptom onset showed no structural abnormalities despite a clinical picture highly suggestive of PRES. In Case 2, initial head CT/CTA revealed subtle bilateral low-attenuation changes in the occipital subcortical white matter, compatible with early vasogenic edema, whereas MRI 12 h later no longer demonstrated visible lesions and was interpreted as favorable evolution of the edema. In Case 3, two head CT/CTA examinations during the acute phase showed no acute parenchymal lesions, hemorrhage, perfusion deficits, or cervical/intracranial vessel occlusion, and MRI could not be performed because the patient was too hemodynamically unstable; PRES was therefore diagnosed clinically. In contrast, Case 4 demonstrated extensive supra- and infratentorial CT/MRI changes typical of PRES, with vasogenic edema, internal hydrocephalus, and subependymal edema. These cases underscore that, in dialysis patients, PRES may present with classic MRI abnormalities, subtle or transient CT changes, or even normal imaging, particularly early in the course.

### 4.3. Diagnosis

PRES diagnosis lacks universally accepted criteria, as clinical and imaging findings are often nonspecific. It is primarily a diagnosis of exclusion, suggested by a constellation of acute neurological symptoms (such as seizures, headache, visual changes, and altered mental status), blood pressure fluctuations, and, when present, vasogenic edema on MRI. Although acute hypertension is common, it is not required for diagnosis. MRI is generally considered the key tool for confirming PRES in the context of compatible symptoms and potential clinical triggers [4,5], but early or MRI-negative presentations have also been reported in high-risk populations. In our series, PRES was diagnosed as a clinical–radiological syndrome. Cases with normal or nonspecific imaging (Cases 1–3) were diagnosed as PRES based on a typical combination of acute neurological symptoms, severe hypertension, ESRD on dialysis, exclusion of stroke and other structural lesions on CT/CTA, and, where performed, MRI, as well as complete reversibility of symptoms after blood pressure control and dialysis optimization. This approach is consistent with describing early or MRI-negative PRES in similar high-risk populations [26].

In PD patients, diagnosis may be particularly challenging because neurological and metabolic disturbances—such as uremic encephalopathy, electrolyte abnormalities, infections, and hypertensive episodes—are already highly prevalent. This was reflected in our series: in Case 1, the initial suspicion was PD-associated peritonitis; in Case 2, an acute cerebrovascular event was considered; Case 3 was initially managed as hypertensive encephalopathy in the context of sepsis; and Case 4 had a combination of resistant hypertension and headache that could have been attributed to chronic ESRD alone. These overlapping features can mimic or mask PRES, making it difficult to distinguish from other dialysis-related complications. Furthermore, blood pressure variability and nonspecific symptom onset may delay recognition, increasing the risk of missed or late diagnosis. Our experience emphasizes the need for a high index of suspicion for PRES in dialysis patients, especially when new-onset seizures, visual disturbances, or abrupt changes in mental status occur.

### 4.4. Treatment

Treatment of PRES focuses on early recognition and rapid control of the precipitating factors, particularly blood pressure and metabolic disturbances [3,27]. Blood pressure management is central, with recommendations to reduce blood pressure by approximately 25% within the first few hours, while avoiding excessive reductions that might compromise cerebral perfusion. Seizures are common and usually require anticonvulsant therapy, although the optimal drug choice and treatment duration remain unclear [1,4]. When PRES is related to cytotoxic or immunosuppressive agents, dose reduction or temporary discontinuation is often required [4].

In our series, management followed these general principles but also highlighted the specific role of dialysis optimization. All four patients received rapid antihypertensive therapy; seizures in Cases 1 and 4 were treated with anticonvulsants, and metabolic derangements were corrected with intensified renal replacement therapy. In three cases (1, 2, and 4), inadequate volume or solute control on PD prompted a switch to HD, which coincided with improved blood pressure stability and gradual resolution of neurological symptoms. Case 3, who had already transitioned to HD before PRES onset and subsequently developed septic shock, required prolonged intensive care but ultimately recovered fully after more than six weeks of multidisciplinary management.

Overall, all patients demonstrated complete resolution of PRES-related symptoms, with time to improvement ranging from 7 to 43 days across the four cases. This pattern is consistent with prior reports indicating that PRES is fundamentally reversible when promptly recognized and appropriately treated [3,25]. At the same time, the need for temporary or permanent modification of dialysis modality in most of our cases suggests that dialysis adequacy, volume status, and blood pressure control are critical modifiable factors in the prevention and management of PRES among PD patients.

From a clinical perspective, our findings underline several practical points for nephrologists caring for PD patients. First, PRES should be actively considered in PD patients with acute neurological symptoms and severe hypertension, even when initial CT or MRI is normal. Second, early neuroimaging (with CT for exclusion of hemorrhage and, when feasible, MRI for the detection of vasogenic edema) can help differentiate PRES from stroke or uremic encephalopathy. Third, reassessment of volume status, dialysis adequacy, and the need for temporary or permanent transition to HD may be crucial components of PRES management in this population.

### 4.5. Limitations

This report describes a small number of patients from a single center, and the observations may therefore not reflect the full spectrum of PRES in dialysis populations. In addition, brain MRI was not available or feasible in all cases, reducing the ability to fully characterize the radiological spectrum of PRES in this cohort. Despite these constraints, the series illustrates several recurring clinical patterns and dialysis-related issues that are directly relevant to the recognition and management of PRES in peritoneal dialysis patients. Future studies should focus on larger, preferably multicenter cohorts with standardized diagnostic criteria, systematic neuroimaging, and longer follow-up to better define risk factors specific to PD, refine blood pressure and volume targets, and evaluate preventive strategies such as PD prescription modifications or early modality switch.

## 5. Conclusions

PRES in patients on peritoneal dialysis is uncommon but clinically important, as its presentation can be easily obscured by pre-existing neurological and metabolic disturbances. In this four-case series, all patients developed PRES in the setting of marked hypertension and uremia, often accompanied by infection, volume overload, or inadequate solute clearance. Neuroimaging findings ranged from normal studies or subtle posterior white matter changes to extensive supra- and infratentorial vasogenic edema, reflecting the heterogeneity of PRES in dialysis patients and the possibility of normal imaging early in the course.

All patients experienced complete resolution of PRES-related symptoms following timely recognition, aggressive blood pressure control, seizure management when required, and optimization of renal replacement therapy, including temporary or permanent transition from peritoneal dialysis to hemodialysis. These observations highlight the need for vigilant blood pressure and volume management, careful assessment of dialysis adequacy, and a low threshold for brain MRI in peritoneal dialysis patients presenting with acute-onset seizures, visual disturbances, or altered mental status.

PRES seems to be much more complex than only manifestations of dialysis treatment. Fluid overload, severe hypervolemia, and high-load uremic toxins result in hypertensive crises with symptoms typical of PRES in this population. Also, there is currently no direct comparative data regarding the incidence of PRES between hemodialysis and PD patients. Although PD may offer a protective effect due to more stable hemodynamic status, it is frequently associated with fluid overload, which often leads to poorly controlled hypertension.

## Figures and Tables

**Table 1:** Dialysis data and medicaments used by the patients.

Case	Age	Sex	Type of PD	Duration of PD	Membrane Status	Dialysis-Related Complications	Laboratory Findings	Antihypertensive Agents
1	28	F	APD	15 months	CC 37.6; Kt/V 1.43; LA	Suboptimal uremia control	Creatinine 1497 mkmol/L Urea 25.4 mmol/L	Bisoprolol Amlodipine Ramipril
2	20	F	APD	11 months	CC 69; Kt/V 1.79; HA	Catheter dysfunction previously	Creatinine 1212 mkmol/L Urea 23.7 mmol/L	Amlodipin Doxazosin Metoprolol Moxonidine Ramipril
3	32	M	CAPD	1.5 years on PD and then 2 months on HD	CC 75; Kt/V 1.9; LA	HD catheter infection at the start of PRES	Creatinine 1075 mkmol/L Urea 15.8 mmol/L	Moxinidine Nifedipine Nebivolol Doxazosine Olmesartan Amlodipin Rilmenidin Torazemide Spironolact
4	41	M	APD	1 month	Not measured	Hypervolemia	Creatinine 1347 mkmol/L Urea 30.7 mmol/L	Metoprolol Moxonidin Olmesartan Torazemide

APD—automated PD; CAPD—continuous ambulatory PD; CC—creatinine clearance; F—female; HD—hemodialysis; M—male.

**Table 2:** Additional clinical characteristics of four PD-related PRES cases.

Cases	Kidney Disease	Comorbidities	PRES Symptoms	BP (mmHg) at Onset	BP (mmHg) at Recovery	MRI/CT Findings	Days to PRES Resolution
1	IgA nephropathy	Arterial hypertension; hypertensive cardiopathy	Altered consciousness Disorientation Generalized seizure Psychosis	150/90	123/70	Brain MRI without visible lesions (PRES diagnosed clinically)	28
2	Alport syndrome	Arterial hypertension	Bilateral blurred vision Headache Nausea and vomiting	200/120	140/102	Subtle bilateral occipital subcortical hypodensities on CT; MRI 12 h later without visible lesions (resolving edema)	7
3	Juvenile nephronophthisis	Arterial hypertension	Weakness Headache Generalized body pain Hand numbness Delirium Disorientation Seizure	210/116	125/80	Two head CTs without acute lesions; MRI not performed (hemodynamic instability)	43
4	Primary focal segmental glomerulosclerosis	Arterial hypertension	Occipital headaches Dizziness Difficult to control hypertension Seizure Blurred vision	187/114	155/90	Supra- and infratentorial PRES-like vasogenic edema (frontoparietal, occipital, and cerebellar), small left frontal microhemorrhage, internal hydrocephalus, and subependymal edema	22

## Data Availability

The original contributions presented in this study are included in the article. Further inquiries can be directed to the corresponding author.
